# Zeolite protects mice from iron‐induced damage in a mouse model trial

**DOI:** 10.1002/2211-5463.12477

**Published:** 2018-10-06

**Authors:** Xiyong Fan, Chris McLaughlin, Jason Ravasini, Cleo Robinson, Anthony M. George

**Affiliations:** ^1^ Faculty of Science School of Life Sciences University of Technology Sydney Broadway New South Wales Australia; ^2^ School of Biomedical Sciences University of Western Australia Crawley Perth Australia; ^3^ Molecular Anatomical Pathology PathWest Laboratory Medicine QEII Medical Centre Nedlands Perth Australia

**Keywords:** iron overload, mouse model, zeolite

## Abstract

For centuries, zeolites have been used for their utility in binding metals, and they feature in a multitude of agricultural and industrial applications in which the honeycombed zeolite structures form ideal ion exchangers, catalysts and binding agents. Zeolites are currently in a transition period, moving towards implementation in human ailments and diseases. Here, we postulated that zeolites may be able to counter the effects of excess iron and conducted a mouse model trial to gauge the utility of this notion. We used the transgenic mouse strain MexTAg299 for a thirty‐week pilot trial in which iron polymaltose and/or the zeolite clinoptilolite was injected into the peritoneum twice weekly. Mice were sacrificed at the end of the trial period and examined by postmortem and histology for significant physiological differences between mouse subgroups. In this study, we demonstrated that a common zeolite, clinoptilolite, is able to maintain the general health and well‐being of mice and prevent iron‐induced deleterious effects following iron overload. When zeolites are given with iron biweekly as intraperitoneal injections, mice showed far less macroscopic visual organ discoloration, along with near normal histology, under iron overload conditions when compared to mice injected with iron only. The purpose of the present pilot study was to examine potential alternatives to current iron chelation treatments, and the results indicate an advantage to using zeolites in conditions of iron excess. Zeolites may have translational potential for use in cases of human iron overload.

**Abbreviations**
Cb: chabazite
Cl: clinoptilolite
GRAS: Generally Regarded As Safe
H&E: haematoxylin & eosin
i.p.: intraperitoneal

Iron overload disorders in humans are manifest mainly as hereditary (primary) hemochromatosis 1, 2, which results mostly in an accumulation of iron in the forms of ferritin and hemosiderin, which cause solid organ injury, particularly to the liver, heart or pancreas. It is the most common genetic disorder in Caucasians. Hereditary hemochromatosis may lead to pathological conditions, including cirrhosis, weight loss, chronic fatigue, liver disease, hepatocellular carcinoma, joint pain, diabetes and cardiomyopathy 3, 4.

Iron overload has been studied extensively in humans and in many animal models of iron‐induced cancer 5, 6. In one trial 7, male Wistar rats were injected (intraperitoneal) with iron saccharate daily for 5 months and sacrificed 1 year later. Nine of 19 rats had mesothelioma tumours upon postmortem examination, leading the authors to conclude that the gradual release of free iron could induce mesotheliomas. This and other animal model studies 5 have identified iron‐induced free radical generation, lipid peroxidation, DNA damage and carcinogenesis.

Iron is the most abundant metal in the human body and is mostly benignly bound to haemoglobin and other proteins. In excess, or when introduced extraneously, it generates hydroxide radicals and pathways to carcinogenesis 8. A US epidemiological study published in 2008 for peripheral arterial disease patients, phlebotomy twice a year reduced the incidence of visceral cancer by 35% and cancer mortality by 61% in a randomized trial using 1277 patients. The consequences of the introduction of excessive quantities of iron sugar polymer composites have been the subject of repeated investigations 5, 6, 9. These studies focussed chiefly on iron overload disorders in humans and in particular hemochromatosis.

Zeolites have been used for centuries for their utility in binding metals and in many agricultural and industrial processes in which the honeycombed zeolite structures are ideal as ion exchangers, catalysts and binding agents. They are useful as feed supplements and are in a transition period of application to human ailments and diseases. Zeolites are hydrated aluminosilicate volcanic minerals that are rare in nature in being negatively charged. Most zeolites have cubic spheroidal shapes with porous internal spaces or cages that are able to adsorb and sequester and neutralize positively charged ions 10. They are ideally suited to adsorbing heavy metals and small organic toxins and have been used for decades in industrial, agricultural and household processes 11. Naturally occurring zeolites, including clinoptilolite and mordenite, have been shown to be biological benign 12, 13, 14, with mordenite eliciting only minor fibrosis and inflammation in rodents 15. The US FDA has classified zeolites as GRAS (Generally Regarded As Safe), with the sole exception of erionite, which is toxic. It is not clear why erionite is toxic, with the most likely reason being its fibrous shape at the micrometre level, making it more closely resemble asbestos.

In this novel pilot mouse model study, we demonstrated adverse effects of iron given as intraperitoneal injections twice weekly to a transgenic mouse strain, and the moderation of these effects by the common zeolite, clinoptilolite. The beneficial effects of the zeolite cotreatment point to the potential for its use in iron overload disorders and to a wider spectrum of uses, including asbestos‐related diseases in which the release of iron is a major causal link.

## Materials and methods

### Preparation of materials

**Figure 1:**
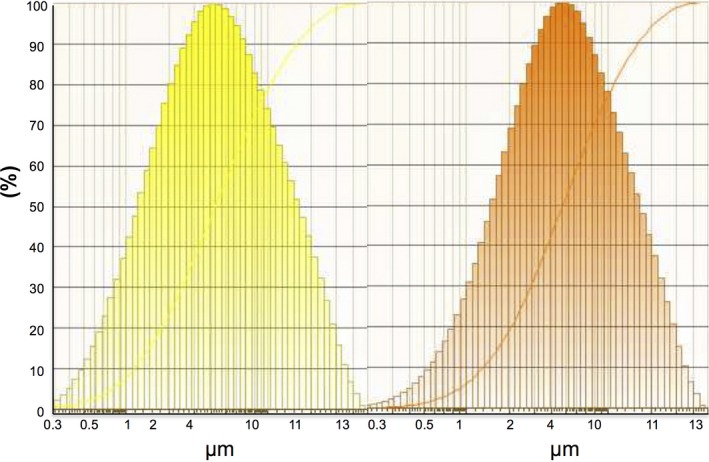
Clinoptilolite (yellow) and chabazite (orange) particle size distributions after milling. The median particle sizes are 4.28 and 5.5 μm, respectively.

Clinoptilolite, a crystalline aluminosilicate zeolite mineral, was obtained from St Cloud Mining Co (NM, USA). Its empirical formula is (K,Ca,Mg) 2O‐AL_2_O_3_‐10SiO_2_‐6H_2_O. The cation exchange capacity is 0.8–1.2 meq·g^−1^, and it is 98% pure by analysis (University of Alberta), but may contain 0.07–0.10% free silica. Over 80% of the particles have diameters of 13.0–25.4 mm. The powder was made into a suspension (1 g zeolite to 1.5 mL water in a 5‐mL capacity grinding bowl with two zircon balls) and milled in a Fritsch Pulverisette 23 for 5 mins. The milled particles were analysed in a Malvern Mastersizer 2000. The median particle size was estimated at 4.28 μm from the particle distribution as illustrated in Fig. 1 (left plot). Chabazite, another zeolite, was milled to a median particle size of 5.50 μm (Fig. 1; right plot). Both milled zeolites had over 80% of the particles in the range of 2 to 13 μm.

### Iron‐binding capacity of zeolites

Atomic absorption spectrometry (AAS) was used to measure the ability of the zeolites, clinoptilolite or chabazite, to chelate iron in a cell‐free format. Iron as FeCl_3_ was diluted in water to a final concentration of 50 μm and zeolites to final concentrations of 1–10 mg·mL^−1^. Solutions or suspensions of ferric chloride standards and clinoptilolite were prepared in sterile deionized water. Samples (*n* = 5) of ferric chloride and clinoptilolite or chabazite were mixed in sealed tubes and placed on a rocking platform for 24 h. Samples were then centrifuged to remove iron–clinoptilolite complexes, and the supernatants were aspirated to fresh tubes and analysed for [Fe^3+^] in an atomic absorption spectrophotometer along with [Fe^3+^] standards.

### Mice

The transgenic mouse strain MexTAg299 h 16 was developed at the School of Medicine & Pharmacology & Western Australian Institute for Medical Research, University of Western Australia. This strain was selected for this iron trial, as a prelude to planned asbestos trials with the same strain. The trial was conducted under guidelines set by the Institutional Animal Care & Ethics Committee, with defined humane endpoints. However, none of the mice reached an endpoint that required euthanasia over the 30 weeks of the trial, but at its conclusion, all mice were euthanized with carbon dioxide.

Targeted expression of the TAg transgene causes mesothelioma to develop more rapidly after asbestos exposure with 100% incidence within 20 to 40 weeks in MexTAg mice compared to 30% incidence in wild‐type mice in 50 to 100 weeks. MexTAg transgenic mice express the SV40 large T antigen specifically in mesothelial cells by use of the cell type‐specific mesothelin promoter. MexTAg mice exposed to asbestos develop cancer that faithfully replicates key features of the pathogenesis of human mesothelioma 17.

### Injection protocol and trial parameters

Iron polymaltose (Ferrosig) and clinoptilolite (St Cloud Mining Co, NM, USA) were prepared in sterile saline (0.9%) for intraperitoneal injection at dosages of 15 mg·kg^−1^ and 45 mg·kg^−1^, respectively, and at 10 mL·kg^−1^. Mice were injected twice weekly with normal saline (control), iron polymaltose (hereafter abbreviated to iron), clinoptilolite or iron + clinoptilolite (Table 1). For the combined injection of iron + clinoptilolite, working solutions of each were prepared separately in saline and mixed in the syringe just prior to peritoneal injection. This protocol was considered more suitable than separate injections of iron and clinoptilolite in the combined treatment mouse subgroup, as only half as many injections were required over the trial period and this was less stressful to the well‐being of the mice. In addition, the iron was complexed as iron polymaltose and therefore would not be immediately bioavailable for sequestration by clinoptilolite 18. The concentration of iron for injection was similar to that used in other mouse and rat trials 5, 6. The clinoptilolite concentration was chosen somewhat arbitrarily at threefold higher than the iron concentration, but well below amounts used (300 mg·kg^−1^) in mouse and dog trials that have shown no deleterious effects over a similar trial period 19, 20.

**Table 1:** Postmortem summary of MexTAg mouse trial

Mouse ID:gender	Body weight (Day 0)	Treatment	Observations			
			Liver	Spleen	Peritoneum	Other Comments
xa01:f	21.5	Saline	Slightly pale			
xa02:f	21	Saline				
xa03:f	22	Saline				
xa04:m	28	Fe	Enlarged		Yellow/orange	Less active
xa05:m	27.2	Fe	Enlarged	Protruding	Yellow/orange	Nodule (abdominal wall)
xa06:m	24	Fe	Very enlarged		Yellow/orange	Less active
xa07:m	27.6	Fe	Enlarged		Orange	Less active
xa08:f	19	Fe	Enlarged		Yellow/orange	
xa09:f	17.4	Fe	Slightly enlarged		Yellow/orange	Small nodule (mesentery)
xa10:f	19.8	Fe	Enlarged	Enlarged	Yellow/orange	Less active
xb01:f	17.1	Zeolite	Slightly enlarged	Enlarged	Zeolite deposit	
xb02:f	21.8	Zeolite			Zeolite deposit	
xb03:f	20.1	Zeolite	Slightly pale	Zeolite deposit	Zeolite deposit	Zeolite deposit
xb04:m	23.2	Zeolite + Fe				Zeolite deposit
xb05:m	22.5	Zeolite + Fe				Zeolite deposit
xb06:m	27	Zeolite + Fe			Pale yellow	
xb07:f	19.5	Zeolite + Fe				Zeolite deposit
xb08:f	19.5	Zeolite + Fe	Zeolite deposit			Zeolite deposit
xb09:f	17.3	Zeolite + Fe				Zeolite deposit
xb10:f	20.3	Zeolite + Fe				Zeolite deposit

A total of 20 MexTAg299 h mice were used in the trial (Table 1), three each as controls (saline or clinoptilolite) and seven each for iron and iron + clinoptilolite injections. The mice were of mixed gender with some siblings and were 65–90 days old when received. They were fed a basal diet and given water *ad libitum*. After a one‐week acclimatization, twice‐weekly injections of saline, clinoptilolite, iron polymaltose, and iron polymaltose and clinoptilolite were given intraperitoneal and the mice were weighed and observed for well‐being or illness on a daily basis. The duration of the trial was 30 weeks. Institutional ethics and biosafety approvals were given ahead of the trial.

### Postmortem

Visual examinations of the gut cavities and tissues and organs were made and recorded. Organ samples were taken for histological staining. A full summary of the postmortem observations is given in Table 1.

### Histology

Sectioned tissues were stained routinely with haematoxylin and eosin (H&E) or Prussian blue. The latter stain was used to identify iron within liver stellate macrophages (Kupffer cells) that forms part of the mononuclear phagocyte system. These sections were taken from the outer edges of the liver. The liver tissues were fixed, sectioned and stained immediately after autopsy. For each section from the iron‐ and iron + zeolite treated mice, ten consecutive fields were chosen to count Kupffer cells with iron deposits. All cells characterized were identified as Kupffer cells by morphology, and although hepatocytes were also present in the sections, these were clearly of different morphology.

### Statistics

Statistical analysis was performed using SPSS 19.0 and GraphPad Prism 7.0a software. The unpaired t‐test or two‐way ANOVA used to analyse differences in the mouse trials for organ cell counts and body weight changes over time.

## Results and Discussion

Natural clinoptilolite was used in this study. Another natural zeolite, chabazite, was included in the initial stages for comparison of milling and iron‐binding capacity. The two zeolites were milled to mean particle sizes of 5.7 and 5.5 μm, respectively (Fig. 1), which we determined to be ideal, in being large enough to prevent most particulates crossing cell membranes, but small enough to minimize innocuous aggregation and immobility.

**Figure 2:**
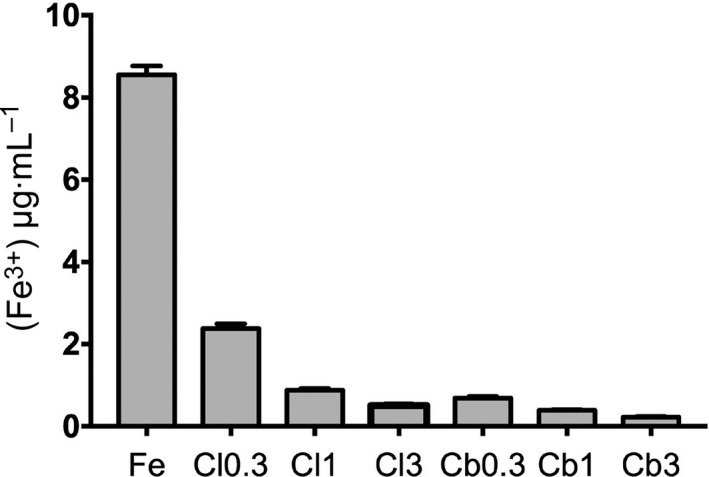
Iron‐binding capacity of clinoptilolite and chabazite *in vitro*. Suspensions of clinoptilolite or chabazite in 50 μm FeCl_3_ solution were mixed for 24 hrs, and residual [Fe^3+^] was measured by atomic absorption against the iron solution as control (50 μm; leftmost column). The three concentrations of the zeolites were 0.3, 1.0 and 3.0 mg·mL^−1^ in water. Free iron was measured from a standard curve. The plotted vertical bars in the figure represent the means of five samples (*n* = 5); and errors as SD.

The iron‐binding capacities of clinoptilolite and chabazite were determined by AAS (Fig. 2). The results plotted in Fig. 2 show that the milled zeolites were able to absorb/chelate large amounts of free iron from solution, a fact already known 10, but that was still required to validate the zeolites for this study. Chabazite adsorbed marginally more iron than clinoptilolite in the concentration range used. Although not directly applicable to the mouse trial in this study, it was expected that the injected clinoptilolite would absorb much of the free iron from the iron polymaltose injections in the subsequent mouse model trial.

**Figure 3:**
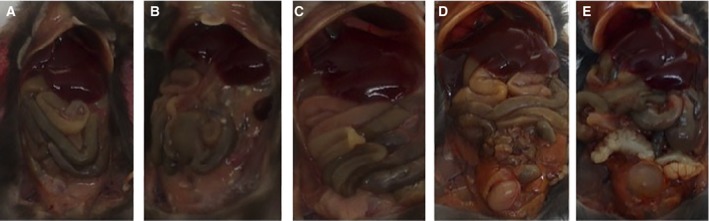
Postmortem examination of the abdominal cavities of mice representative of each subgroup. Order is as follows: (A) saline; (B) zeolite; (C) zeolite plus iron; (D) iron (male); (E) iron (female).

The mouse model trial was set up for 30 weeks of duration using four subgroups of the MexTAg299 h transgenic strain, to be injected intraperitoneal twice weekly with saline, iron, clinoptilolite or iron + clinoptilolite (Table 1). Postmortem macroscopic observations of the mice were different, in that the mice injected with saline, zeolite or iron + zeolite looked normal in coloration (Fig. 3A–C), whereas mice injected with iron showed the gut and organs to be significantly yellow/orange in coloration, especially in the lower abdomen (Fig. 3D,E). These representative photographs were typical of all mice within the four subgroups, with the sole exception that one of the iron + zeolite mice had a slightly yellowish coloration in the lower abdomen cavity. Our conclusions were that the coloration was due to the deposition and therefore lack of clearance of iron. In contrast, in the iron + zeolite mice, much of the iron was sequestered as inert or innocuous within the zeolite particles, based on the fact that there was no orange coloration of the abdominal cavity in the macroscopic postmortem examinations of the iron + zeolite mice. Further evidence for this was derived from the histology (see below).

A more detailed macroscopic examination revealed further features of the effects of iron on the abdominal organs (Table 1). In the mice injected with iron, all showed enlarged livers and two of seven mice had enlarged spleens. None of the control or iron + zeolite mice showed any enlargement of organs visible to the naked eye (Table 1). There were some zeolite aggregates within the abdominal cavity, but there was no evidence that these deposits affected the well‐being of the mice, which is in keeping from the results of earlier studies in which mice were given intraperitoneal injections of ten times higher amounts of clinoptilolite with no adverse behavioural effects in the mice 20, 21.

Four of the seven iron‐treated mice were noticeably less active in the latter third of the trial than all other mice. Two of the iron subgroup had small nodules in the abdomen and these were surmised to be granulomas rather than tumours, in view of these mice not showing any of the telltale symptoms of tumour growth, notably as marked physical changes in appearance or behaviour, or abdominal tumours and/or blood‐stained ascites fluid. These observations are in keeping with previous iron overload mouse or rat studies have shown that tumours are rarely seen inside 12 months 22, 23, whereas the appearance of granuloma nodules was more common. A similar outcome was observed in a large and long‐term (2.5 years) rat trial in which cellulose fibres were injected at regular intervals into the peritoneal cavity 24. Only 14 of 200 rats developed malignant tumours and none earlier than 18 months, whereas many granulomas and fibrous adhesions were detected in many of the rats.

**Figure 4:**
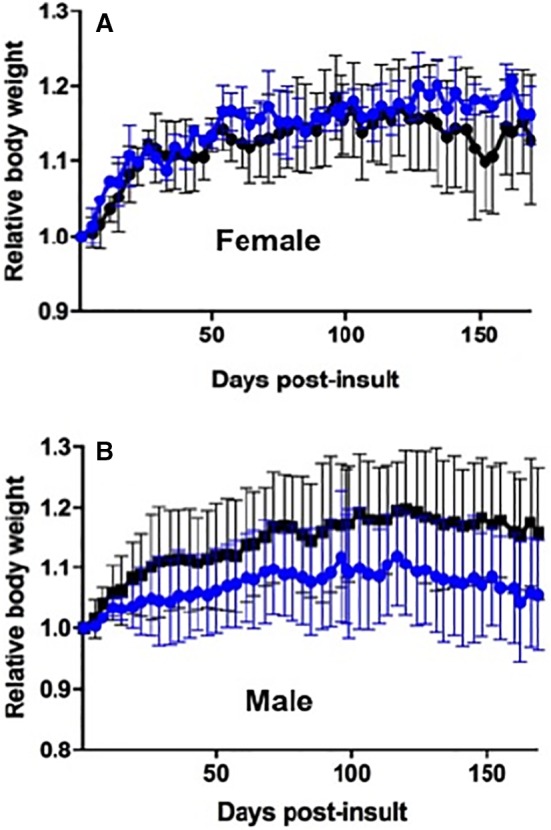
Body weight changes over time in female and male mice injected intraperitoneal with iron only (blue traces; *n* = 3) or iron plus clinoptilolite (black traces; *n* = 4 female, *n* = 3, male). The differences between the iron and iron plus clinoptilolite treatments are only significant for the male body weight traces (*P* < 0.0001). A control trace for female mice (*n* = 3) treated with clinoptilolite alone is not shown, but it is approximately similar to the black trace in (A). All errors as SD.

**Figure 5:**
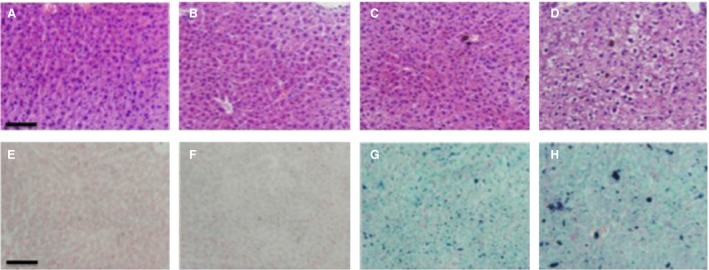
Histology of liver sections. Top and bottom plates are H&E and Prussian blue stained, respectively. Saline (A,E); zeolite (B,F); zeolite plus iron (C,G); iron (D,H). The black bar at left bottom in the first panel of each row represents 80 μm.

A comparison of the mouse weights across the trial showed no significant difference among female mice injected with iron only or with iron plus clinoptilolite (Fig. 4A), but there was a significant difference for the same treatments in male mice (Fig. 4B), where lower body weights were registered almost from the start of the trial to its completion in the iron only subgroup. We surmise that the gender differences are real in terms of the lower body weights of male versus female mice treated with iron alone, based on previous observations that female mice are more tolerant of iron overload 25, 26. In contrast to the iron subgroups, there were virtually no differences in body weights of males versus females in the iron plus clinoptilolite subgroups. This result is supported by the postmortem examinations and histology (Table 1; Figs 3 and 5), which showed little, if any, differences between male and female mice treated with iron plus clinoptilolite. Lastly, a clinoptilolite control subgroup of three female mice showed body weights near those of the doubly treated male and female mice subgroups (not shown).

There was a small significant difference between the zeolite + iron and female + iron subgroups (*P* = 0.0013). All other pairings were significantly different (*P* < 0.0001). Interestingly, the female + iron and male + iron groups were at opposite ends of the body weight gain plots, with the zeolite control and zeolite + iron plots between the other two. It does seem that the female mice (collectively) were beginning to gain more weight than normal, which might have been more pronounced were the trial to have run longer. On the other hand, the iron‐treated male subgroup showed marked and significant differences in weight gains to all other subgroups (Fig. 4; red trace). This trend may be explained by observations elsewhere that male mice and rats are much more susceptible than are females to cancer induction by iron complexes; and the incidence of cancer is paralleled by increased lipid peroxidation. The hormonal environment is responsible for this sensitivity 5. In summary, the weight gain trends cannot be taken alone as indicators of the iron overload and zeolite effects, but their significance becomes more pronounced when the other parameters (postmortems and histology) are included.

The liver histology supported the earlier data. Although Kupffer cells may migrate within the liver, they are sensitive to harmful or toxic agents such as iron, and this results in the localization of more of the Kupffer cells in the outer liver peritoneal layer, where we took sections for haematoxylin and eosin (H&E) and Prussian blue staining. The H&E‐stained sections show normal cellular physiology from the liver peritoneum of saline, zeolite and iron + zeolite mice (Fig. 5A,B,C), but clear differences in the tissue from the iron subgroup, in which the liver cells depict swelling and disorganization (Fig. 5D). The Prussian blue stain was used to identify iron particles within liver stellate macrophages (Kupffer cells) that forms part of the mononuclear phagocyte system. Here, it is clear that the iron‐treated mice retained more iron aggregates in liver cells (Fig. 5H) than did cells from the iron + zeolite treated mice (Fig. 5G). Although only single sections are depicted, similar histology was observed in all mice in each subgroup. Saline and zeolite liver sections were also stained with Prussian blue, but there was no visible iron staining (Fig. 5E,F).

All control mice (saline or clinoptilolite) and the iron + zeolite subgroup appeared normal to the end of the trial, and postmortem and histology confirmed that the tissues and cell morphology were essentially indistinguishable. However, there were clear differences between these mice and the seven iron‐treated mice, with postmortems showing yellowing of the tissues and enlarged organs in the peritoneum. Liver histology showed damage to liver tissue in iron‐treated mice only. Extensive deposition of iron was seen in the Kupffer cells in iron‐treated mice, but much less iron in the iron + zeolite subgroup.

**Figure 6:**
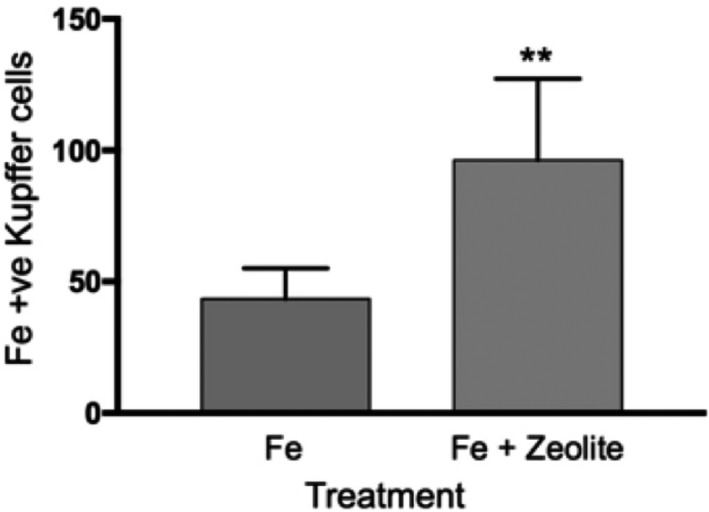
Phagocytosed iron deposits in liver Kupffer cells in iron‐treated mice (left bar) and iron + zeolite treated mice (right bar). The differences were analysed using an unpaired t‐test, which calculated the difference as significant (*P* < 0.0012). Errors as SD. **indicates *P* < 0.01.

Finally, there were differences in the uptake of iron by the liver macrophage (Kupffer cell) system. The number of iron‐containing macrophages seen in iron + zeolite sectioned liver was about double the macrophage total counted in iron‐treated mice, an indication of significantly more successful clearance of iron by higher numbers of macrophages in mice cotreated with zeolite (Fig. 6; right bar). This result supports the Prussian blue histology depicted in Fig. 5, in which the accumulation of iron was much less prevalent in sections from the iron + zeolite treated mice (Fig. 5G) than in the iron‐treated mice (Fig. 5H). There were a small number of lymphocytes in the histology slides from the zeolite and iron + zeolite subgroups (not shown).

The immunostimulatory effects of zeolites are well documented. Clinoptilolite has been shown to have an immunostimulatory effect in mice 21, 27. In the first of these studies, mice injected intraperitoneal with clinoptilolite caused an increase in the number of peritoneal macrophages, a similar result to what we observed (Fig. 6). In the latter study, micronized zeolite was administered by gastric intubation to mice injected with melanoma cells. The number of melanoma metastases and the degree of lipid peroxidation decreased. The authors suggest a possible mechanism for these results is an increased activation of macrophages and stimulants of the immune system. Other studies have found that orally administered clinoptilolite is a potent antioxidant 28 and does not alter serum chemistry to any significant extent 29.

Cell death is crucial for normal development, homeostasis and the prevention of hyperproliferative diseases such as cancer 30, 31. Iron is an important contributor to cell damage and death in humans with iron overload disease. Ferroptosis is a specific iron‐dependent form of nonapoptotic cell death, and it is morphologically, biochemically and genetically distinct from apoptosis, necrosis and autophagy 32. Although ferroptosis is characterized by lipid peroxidation and reactive oxygen generation from iron Fenton chemistry, the precise role of iron remains unclear. It may involve one or more iron‐dependent enzymes functioning as part of a core oxidative lethal mechanism.

Many animal models of iron‐induced cancers including mesothelioma have been studied 5, 6, and these trials were typically longer than the present study. In these trials, the only treatment considered was that of the reference standard iron chelator deferoxamine. However, having been used clinically for over four decades, its effectiveness is limited by a demanding therapeutic regimen of slow subcutaneous administration and poor plasma half‐life that leads to poor compliance 33, 34.

Deferasirox (Novartis Pharma AG) represents a new class of tridentate iron chelators, which can be given as an oral daily dose. It has been in use since 2005, but warnings about gastrointestinal haemorrhage and kidney and liver failure were attached to its use following a report of 1320 patient deaths by the Institute of Safe Medical Practices in 2009. In contrast to chelators with poor patient outcomes, zeolites are natural, inert and nontoxic aluminosilicates, which are classified as GRAS (Generally Regarded As Safe) by the US Food and Drug Administration. One study 35 found that clinoptilolite (the same zeolite used in our study) was inert in the rat lung at 360 days. Others have shown that clinoptilolite has no known carcinogenic effect 15, and only very high doses (300 mg·kg^−1^) of clinoptilolite cause a focal storage type reaction in lung tissue 36. This concentration (300 mg·kg^−1^) is ten times the amount used in our study.

The purpose of the present study was to examine potential alternatives to current chelator treatments of iron overload disorders, and the results suggest that common zeolites may open a new treatment potential for iron overload disorders.

This pilot study served as a prelude to a broader mouse model study, using the same MexTAg299 h strain to test the effectiveness of clinoptilolite to scavenge iron released from asbestos injected into the peritoneum, and thereby moderate the onset of mesothelioma, which has been found to indeed be the case (manuscript in preparation). The very promising findings from this asbestos/mesothelioma study reinforce the importance of the present iron mouse model study, as the sequestration of iron is one of the mechanisms by which the moderation of mesothelioma has been postulated in the asbestos mouse model.

## Author contributions

XF helped design and performed the mouse trial, postmortems and histology; and helped with the writing of the manuscript. CM performed the zeolite microfining and iron‐binding capacity work and helped write some of the methods. JR was involved in the planning and follow‐up of intellectual input into the study and manuscript. AMG helped design the study and assisted in some of the experimental work. He wrote the manuscript.

## Conflict of interest

The authors declare no conflict of interest.
